# Leveraging automotive fuel cells can supply zero-emission peak power in the near-term

**DOI:** 10.1016/j.isci.2024.110246

**Published:** 2024-06-11

**Authors:** Emilia Chojkiewicz, Amol Phadke

**Affiliations:** 1 Energy Markets and Policy Group, Energy Analysis and Environmental Impacts, Energy Technologies Area, Lawrence Berkeley National Laboratory, Berkeley, CA 94720, USA; 2 Goldman School of Public Policy, University of California, Berkeley, Berkeley, CA 94720, USA

**Keywords:** energy systems, energy storage, economics

## Abstract

An increasingly decarbonized yet resilient power grid requires the corresponding build-out of dispatchable zero-emission resources to supply peak power. However, there is a recognized dearth of solutions which can serve multi-day peak demand events both cost-effectively and with near-term deployability. Here, we find that pairing low-cost automotive fuel cells with hydrogen storage in salt caverns can serve as a peaker plant at less than 500 US$/kW at present, a fraction of the cost of conventional fossil fuel-fired peakers. We demonstrate the peaker’s value for long duration storage by comparing it with pumped hydro and assessing its profitability within Texas’ energy-only market region. Although deployment of these peakers is constrained by the presence of salt caverns, we show that a number of sites in the United States and Europe are endowed with suitable salt formations, while utilizing hydrogen storage in pressurized containers could form a location-agnostic peak power solution.

## Highlights

• Pairing automotive fuel cells with salt cavern hydrogen storage can serve as a peaker
• The peaker costs less than 500 US/kW, competitive with conventional gas peakers
• A number of sites across North America and Europe have suitable salt formations
• There is near-term, GW-scale potential for deployment of zero-emission peak power

## Graphical abstract

**Figure:**
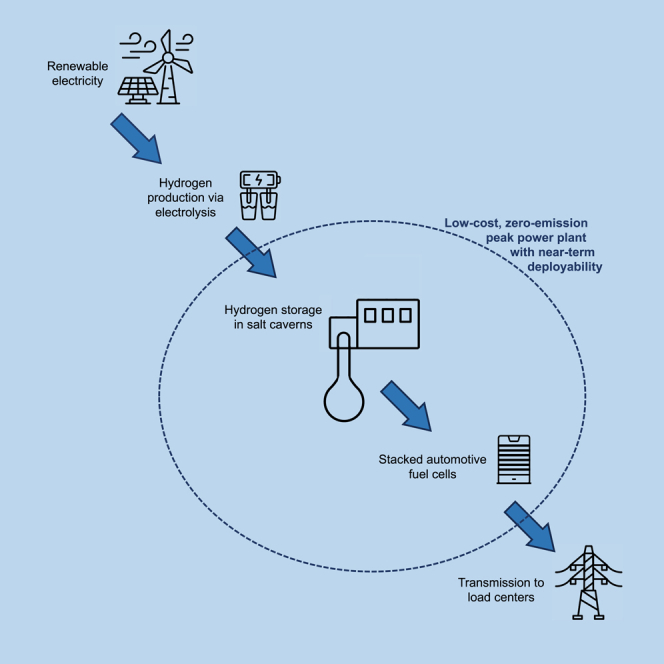

## Introduction

Amid a growing penetration of intermittent renewable generation, the electric power system will increasingly require dispatchable zero-emission resources to serve periods of high demand and/or low renewable supply. Today’s grid operators typically meet this need by dispatching fossil fuel-fired “peakers”, units which sit idle the majority of the time but can quickly ramp up to balance fluctuations in demand. While peakers typically operate at a maximum capacity factor of approximately 10%,^1^ they play a key role in ensuring resource adequacy—the ability of the electricity system to meet consumer demand under all operating conditions.^2^ However, these plants predominantly burn natural gas (NG) or oil, emit high rates of pollutants per megawatt-hour of generated electricity, and are disproportionately located in disadvantaged communities.^1^^,^^3^^,^^4^ Some jurisdictions are retiring their aged and inefficient peaking assets in order to meet climate targets, although this strategy risks jeopardizing operating reserves and resource adequacy.^2^ Simultaneously, the magnitude of this need is steadily increasing with the growing electrification of the industrial, transportation and residential sectors; on the path to net zero emissions by 2050, global electricity demand is forecast to rise by 40% by 2030 and by over 250% by 2050.^5^ The United States (US) alone is estimated to require over 300 GW of peak generation capacity by 2035 to accommodate the 50–150% increase in peak demand.^6^^,^^7^ A crucial part of economy-wide decarbonization, therefore, involves not only the identification of climate-friendly solutions for peak power supply, but also the deployment of large peak power capacity that is set to be over double that of today’s.

Whereas the challenge of peak power supply in a decarbonized power system has traditionally been viewed as a distant concern rather than a near-term issue, the urgency of the need has been underscored by the rising uncertainty and repercussions from increasingly frequent and severe extreme weather events. In recent years, the power system has been threatened by droughts, heat waves, wildfires, blizzards, and other natural disasters, which have incapacitated both generation and transmission resources and led to operating reserve shortfalls.^8^ The extreme nature of these events often simultaneously elicits high electricity demand, exacerbating supply risks as demonstrated by Winter Storm Uri in Texas in 2022^9^ and increasingly record-breaking heat waves in California^10^ and across Europe in recent summers.^11^ With climate change expected to worsen the impacts of many extreme weather events in the future, power system planners are seeking sustainable strategies to uphold grid reliability and resilience.

Energy storage emerges as an attractive option for addressing this challenge since it can provide dispatchability, fast ramping speeds as well as ancillary services. However, there is a recognized dearth of technological solutions in the 10–100 h duration category: for these multi-day peak demand events, current lithium-ion batteries cannot provide the necessary duration for cost-effective arbitrage while seasonal energy storage like pumped hydro storage (PHS) is limited in geographic scope beyond its present-day reach. Previous policy targets and initiatives aimed to overcome these limitations^12^ have reaped little success, with other recognized long duration energy storage (LDES) technologies—such as iron air batteries, thermal storage, etc. —remaining commercially unavailable and/or cost-prohibitive.^6^^,^^13^ The prevailing strategies to maintain sufficient peak capacity in a decarbonized power system hinge upon either retaining NG combustion turbines (CTs) with plans to offset carbon emissions, necessitating carbon capture and sequestration on fossil fuel-fired plants, or installing/retrofitting turbines that are capable of running on hydrogen.^14^ However, hydrogen CTs are not currently commercially available at scale, not least without fuel blending, and while they do not emit carbon, they do emit NOx—a precursor to harmful ground-level ozone formation.^15^ Meanwhile, fuel cells (FCs) can convert hydrogen into electricity in an emission-free chemical reaction although their application to power generation has been historically discounted due to assumptions of high costs for the capital investment in stationary FCs as well as hydrogen production.^16^^,^^17^^,^^18^^,^^19^^,^^20^^,^^21^ Nevertheless, given the climate imperative, the identification of LDES alternatives with near-term deployability is particularly pertinent.

Two recent developments have reinvigorated the opportunities for hydrogen and FCs in electricity generation. First, generous tax credits in the Inflation Reduction Act (IRA) have greatly improved the economics of green hydrogen production in the US, i.e., zero-carbon hydrogen production via electrolysis.^22^ Second, as a result of climate pressures and policy incentives to decarbonize the transportation sector, automotive FCs have become widely commercially available: the manufacturing capacity of automotive FCs based on Proton Exchange Membrane (PEM) technology reached approximately 330,000 units/year in 2022.^23^ As a result of this scaling of mass manufacturing as well as lower durability ratings reflecting the FCs’ rated lifetimes,^24^^,^^25^^,^^26^ automotive FCs cost a fraction of stationary FCs which despite similar raw materials have conversely exhibited minimal growth in recent years, largely focusing on combined heat and power (CHP) rather than power-only applications.^18^^,^^23^^,^^27^ However, the infrequency of peak power demand events and the corresponding low annual capacity factor of peaker plants (typically, less than 10%) does not require the higher durability ratings of stationary FCs, typically designed specifically for baseload operation. Although literature has long noted the potential contribution of hydrogen and FCs to serving peak power demand as well as backup power due to their flexibility and controllability,^20^^,^^28^ no study has explored the techno-economics of directly stacking automotive FCs in a stationary application, i.e., without the surrounding vehicle, to serve as a zero-emission, low-cost peaker. Today, the real-world implementation of automotive FCs in a stationary application primarily constitutes demonstration projects by manufacturers^29^^,^^30^^,^^31^^,^^32^ and while previous work notes the possibility,^13^^,^^17^ notable gaps remain in evaluating both the economics of project development and the potential to serve multi-day peak power demand events.

In this article, we assess the techno-economics of leveraging automotive FCs in a stationary application as a zero-emission, low-cost peaker plant. Given the commercial maturity and low costs, we find that an auto FC peaker paired with salt cavern hydrogen storage can provide emission-free peak power at less than 500 US$/kW, cost-competitively to a NG CT in the US today. We demonstrate the value of an auto FC peaker’s ability to operate over multi-day periods by comparing it with the other mainstream alternative, pumped hydro, and assessing its profitability within Texas’ energy-only market (EOM) region. While the affordability of an auto FC peaker rests upon the availability of geologic hydrogen storage in close proximity, we show that a number of sites in the US and Europe are endowed with suitable salt formations, presenting a feasible near-term option for zero-emission peak power, while future developments and falling costs of other hydrogen storage technologies can make auto FC peakers a global emission-free peak power solution.

## Results

### Feasibility of an auto FC peaker

**Figure 1:**
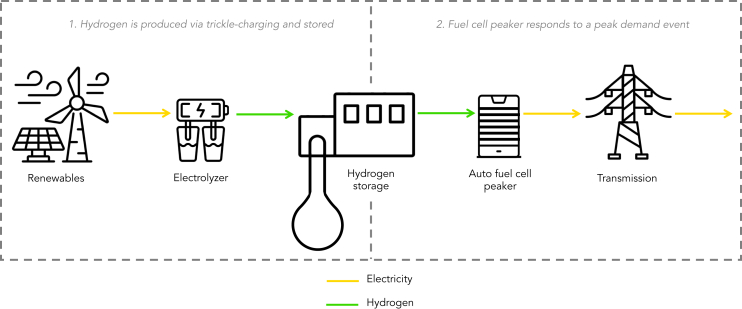
Schematic of an auto fuel cell peaker and its production chain In the first step, hydrogen is produced from renewable energy sources by trickle-charging an electrolyzer - i.e., producing hydrogen during times of abundant renewable electricity generation—and then stored. During a peak demand event (<10% of the hours of the year), the fuel cell peaker pulls the hydrogen from storage to produce green electricity and send it out onto the grid. Yellow lines depict flows of electricity, while green lines represent flows of hydrogen.

To avoid near-term hydrogen transportation barriers such as pipeline construction, trucking, etc., the utilization of green hydrogen in an auto FC peaker necessitates the availability of hydrogen production and storage in close proximity. This way, green hydrogen can be produced via electrolysis during times of abundant renewable electricity generation and stored until it is needed to supply the auto FC peaker during a peak demand event. An overview of the process is shown in Figure 1.

**Figure 2:**
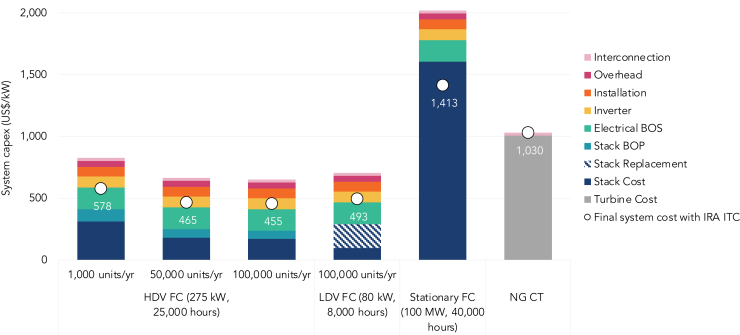
Build-up of peaker system capital costs Bars show the build-up of capital costs for a fuel cell peaker based on annual production rates (in units/year), designed fuel cell capacity and durability ratings, compared to a conventional peaker i.e., a natural gas combustion turbine (see STAR methods and Table S1 for the detailed assumptions). White dots represent the final system capex after the IRA Investment Tax Credit (ITC) of 30% is applied. Assumes a 30-year system lifetime and durability ratings that reflect the rated stack lifetime. Note that the stack cost for both the LDV and stationary FCs reflects both the stack and its BOP, as the cost breakdown was not available. In reality, actual costs may vary not only with production rates but also with designed fuel cell capacity. BOS, Balance of System; BOP, Balance of Plant; IRA ITC, Inflation Reduction Act Investment Tax Credit; NG CT, Natural Gas Combustion Turbine; FC, Fuel Cell; HDV, Heavy Duty Vehicle; LDV, Light Duty Vehicle. 2023 US$.

We first assess the costs of FC peaker investment, leveraging fuel cell stack costs from the US Department of Energy (DOE)^17^^,^^24^^,^^25^^,^^26^ and using utility-scale storage system integration costs from the National Renewable Energy Laboratory (NREL) as a proxy^33^ (see STAR methods and Tables S1 and S2 for the detailed assumptions). Figure 2 shows how a larger scale of manufacturing as well as lower durability requirements contribute to FCs for both light duty vehicles (LDVs) like cars and heavy duty vehicles (HDVs) like trucks being significantly less expensive than counterparts intended for stationary applications as well as conventional NG CTs.^17^^,^^23^^,^^24^^,^^25^^,^^26^^,^^33^^,^^34^ We select HDV FCs for subsequent analysis because their higher durability ratings (on the order of 25,000 h) do not necessitate stack replacement like LDV FCs (with durability ratings of approx. 8,000 h) over a 30-year lifetime of the asset utilized at an annual capacity factor of less than 10%, lowering total capital costs (Figure 2). We assume costs based on an annual production rate of 100,000 systems/year, which represents the same order of magnitude of global PEM manufacturing in 2022 (approximately 330,000 units/year)^21^ and is further supported by the industry shift from LDV to HDV applications in recent years.^35^ With the IRA investment tax credit (ITC) applied, the capex of HDV FCs is less than half that of NG CTs and a third of stationary FCs; even without the IRA ITC, the capex is still about 60% that of NG CTs.^22^

Based on the power requirements of the fuel cell peaker, we then size the green hydrogen production and storage. Assuming that the on-site electrolyzers are trickle-charged from co-located wind and solar generators using US-average capacity factors, we calculate the levelized cost of hydrogen production (LCOH) (see STAR methods and Tables S1 and S2). We assume the process claims two production tax credits (PTCs) from the IRA for renewable electricity production as well as hydrogen production.

**Figure 3:**
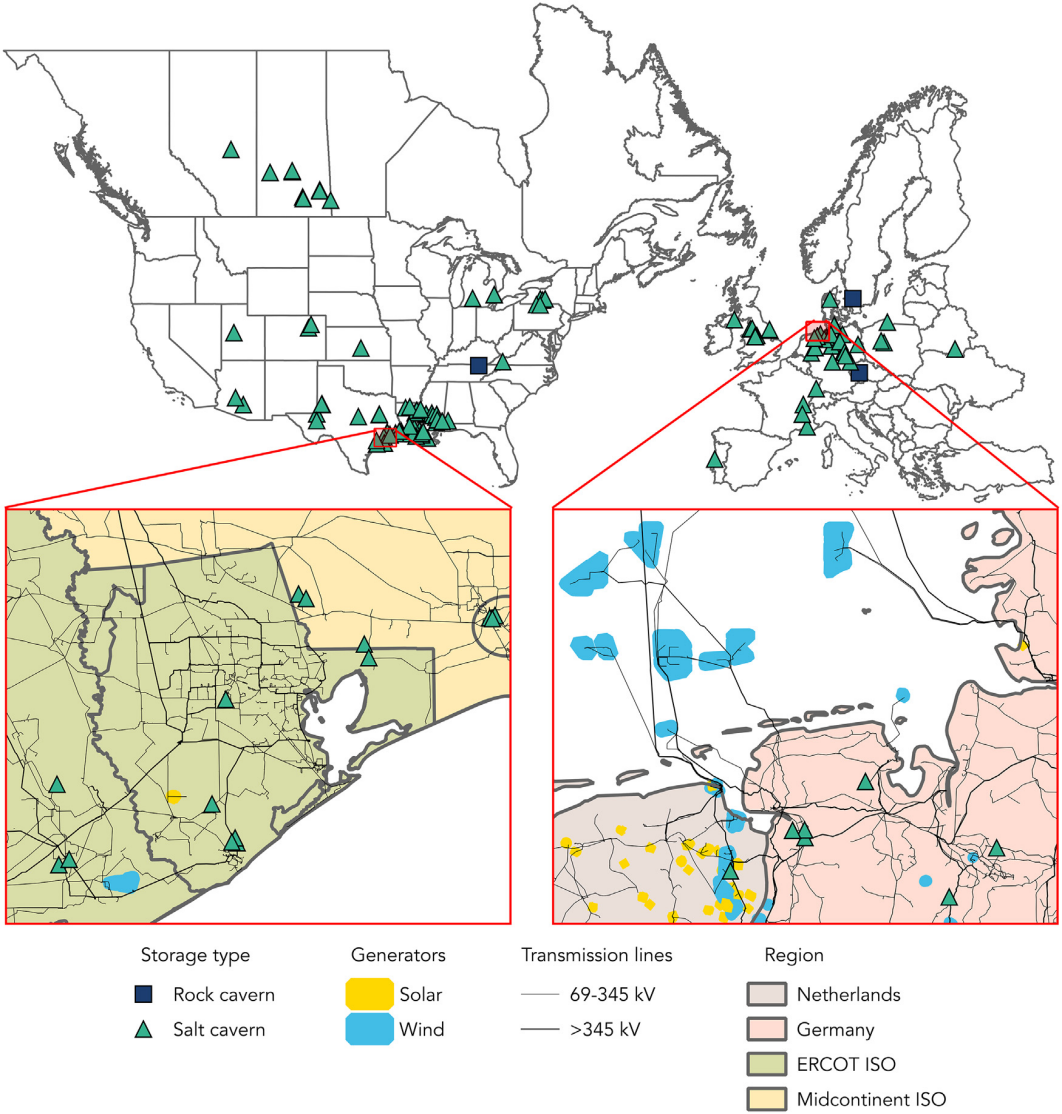
Geographic scope of geologic hydrogen storage in North America and Europe While geologic hydrogen storage is currently limited to six salt caverns in Texas and the United Kingdom,^36^ the tens of sites designated for natural gas storage—including facilities that are in operation, retired, under construction or planned—indicate the known geographic scope of geologic hydrogen storage.^37^^,^^38^ The current opportunity for hydrogen storage in salt caverns is marked by green triangles, while navy squares depict the future opportunity with the technical development of storage in rock caverns.^36^ Enlarged images of the Houston metropolitan area in Texas as well as the coastline of the Netherlands and Germany show that significant geologic hydrogen storage is available in close proximity to existing power transmission infrastructure that brings renewables from west Texas and offshore wind from the North Sea into load centers. Yellow dots show existing solar plants while blue dots show existing wind plants, with a plant output over 10 MW.

Regarding hydrogen storage, the geologic storage of hydrogen in salt caverns is recognized as a mature and cost-effective solution, particularly relevant for applications with low cycling rates and large volume requirements such as peak power supply.^17^^,^^21^ Six salt caverns around the globe have safely stored hydrogen for decades, with tens more used for NG storage, reflecting historical market needs.^36^ The physical and chemical properties of the rock salt mean that salt caverns are airtight and chemically inert; this results in near-zero losses and disbars additional gas purification as may be required for other geologic hydrogen storage options, such as depleted gas fields and aquifers, which carry a risk of contamination with residual gas and/or chemical reactions from microbes.^36^ Appropriate salt formations, including bedded salt and salt domes, are distributed across North America and Europe with high concentrations in the Gulf states and northwest Europe, as indicated by sites designated for NG storage today (Figure 3).^37^^,^^38^ With further surveying of appropriate geologic formations in other global regions, including large power markets as India and China, the known geographic scope of geologic hydrogen storage is likely to be expanded. Further, pending a successful proof of concept of hydrogen storage in lined rock caverns and corresponding technological commercialization, the reach of geologic hydrogen storage may expand further.^36^^,^^39^ Pressurized containers present an alternative commercially available yet location-agnostic storage solution, although at significantly higher costs.^36^^,^^39^ Here, we evaluate an auto FC peaker with both salt cavern storage (SCS) as well as pressurized container storage (PCS) given their high commercial readiness level.

**Figure 4:**
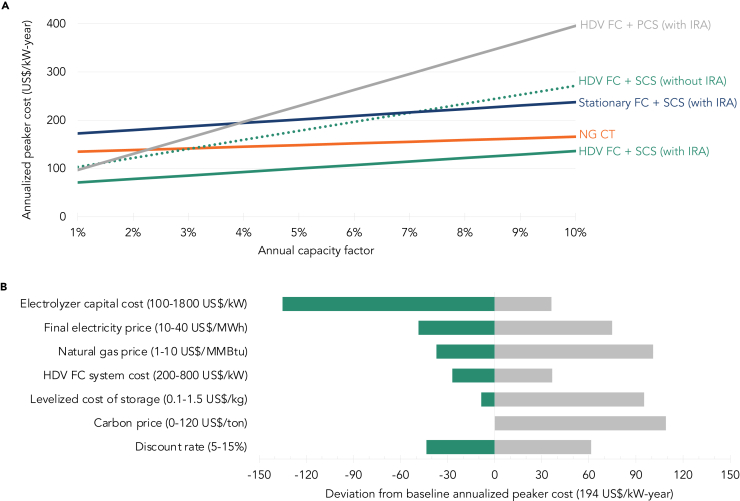
Peaker costs and sensitivities (A) Lines show annualized peaker costs, representing the sum of annualized capex costs as well as operating and fuel costs, as a function of peaker utilization. Steeper slopes of the lines indicate higher operating expenses with each incremental unit of operation. (B) Baseline annualized peaker cost is 194 US$/kW-year, the point at which an HDV FC + SCS peaker breaks even with an NG CT peaker, i.e., a 18% capacity factor. Baseline assumptions include a 1441 US$/kW electrolyzer capital cost, 22 US$/MWh final electricity price, 3.42 US$/MMBtu natural gas price, 455 US$/kW HDV FC system cost, 0.22 US$/kg levelized cost of storage, a 0 US$/ton carbon price, and a discount rate of 10%. For each input, values are calculated as the deviations from the baseline HDV FC and NG CT peaker cost while keeping the baseline assumptions constant for all other inputs, with gray bars depicting an increase in annualized peaker cost while green bars depict a decrease in annualized peaker costs. The natural gas price and carbon price input sensitivities pertain to NG CTs only. HDV, Heavy Duty Vehicle; FC, Fuel Cell; PCS, Pressurized Container Storage; SCS, Salt Cavern Storage; NG CT, Natural Gas Combustion Turbine. 2023 US$.

We calculate the annualized costs of hydrogen production, storage and system investment as a function of peaker utilization (Figure 4A). Stacked IRA incentives play a key role in bringing down both the capital costs and operating expenses (primarily fuel) of an HDV FC + SCS peaker, improving its cost-competitiveness with a conventional NG CT—even without a carbon price. In comparison, despite the inclusion of stacked IRA incentives, a stationary FC + SCS peaker remains significantly more expensive due to its high capex and an HDV FC + PCS peaker remains significantly more expensive due to its high levelized storage costs in this low-cycling application.

We analyze the sensitivity of our results from the baseline HDV FC + SCS with IRA case to changes in electrolyzer costs, final electricity price, NG price, fuel cell system costs, the levelized cost of storage, the inclusion of a carbon price, and the discount rate at the breakeven point with an NG CT peaker, i.e., a 23.5% capacity factor. Figure 4B depicts the range of annualized peaker costs for each input assumption, with the largest price impacts driven by the capital cost of the electrolyzer and the final electricity price. This indicates that the fuel costs associated with hydrogen production, and thus the operating expenses, play a major role in the total costs of an HDV FC peaker rather than the capital costs of the HDV FCs themselves. This is reflective of the typical cost structure of peaker plants, which tend to have low capital costs but high operating expenses due to the uncertainty around their utilization. We also explore the implications of lower production volumes and thus higher fuel cell capital costs, varying the HDV FC system cost up to $800/kW, which represents the approximate cost of an HDV FC system at a small production volume of 1,000 units/year without IRA incentives (Figure 2). Such a capex would only translate to raising the annualized peaker cost by $37/kW-year or approximately 17%, a relatively small increase compared to for example the implications of higher final electricity prices. Meanwhile, the inclusion of a carbon price as well as an increase in NG prices could significantly raise the annualized costs of an NG CT and further strengthen the competitiveness of HDV FC peakers. The variation of the assumed discount rate has a relatively limited impact on the annualized peaker costs. Not shown in the figure is the impact of a lower system life than the assumed 30-year lifetime based on the 25,000 HDV durability rating today (per existing literature and verified by currently available HDV FCs on the market^40^) and an annual capacity factor of less than 10%; however, the present value of a future stack replacement at say 15 years is minimal, also given that fuel cell costs are expected to fall.^24^^,^^25^^,^^26^

### Value comparison to long-duration energy storage alternatives

We next value an HDV FC + SCS peaker against pumped hydro, the only commercially available zero-emission long-duration energy storage (LDES) alternative that could serve a 1–10 days reliability event (e.g., peak demand or renewable drought) and has utility-scale deployment in several power markets across the globe in the present-day. Utilizing the same assumptions as outlined in the Duration Addition to electricitY Storage (DAYS) program^12^—a US Department of Energy (DOE) initiative to lower the costs of LDES—we set the charging electricity price at $25/MWh and consider a range of 10–100 h’ discharge duration at rated power, with the number of events arbitraged per year declining with duration (i.e., a 10-h asset arbitrages roughly 110 events per year, while a 100-h asset arbitrages 12 events) (see STAR methods for additional detail).

**Figure 5:**
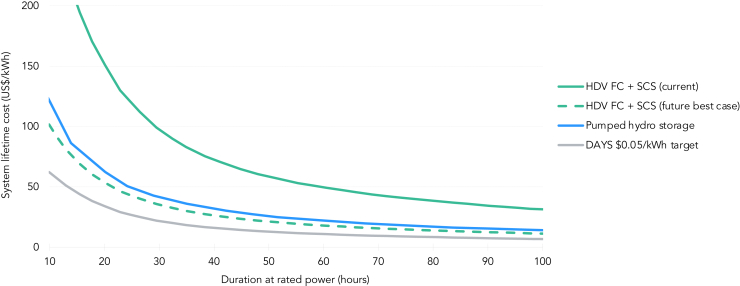
Cost comparison of an FC peaker against pumped hydro storage for different discharge durations Costs of long-duration energy storage options as a function of discharge duration at rated power. Figure include an HDV FC + SCS peaker at present with a solid green line, an HDV FC + SCS peaker in the future best case with a green dashed line, pumped hydro storage at present with a blue line, and the DAYS $0.05/kWh-cycle target with a solid gray line.

Figure 5 shows the levelized cost of the proposed HDV FC + SCS system. While current costs range above pumped hydro, costs in the coming years are projected to fall below pumped hydro over all durations and cycling frequencies. Unlike pumped hydro, however, hydrogen storage systems are not dependent on large-scale water availability, and the locations of salt caverns (Figure 3) tend to differ drastically from the locations of large hydro facilities (predominantly in the northeast and northwest US). Deployment of HDV FC + SCS peakers at scale would therefore increase the geographic scope of long duration storage options. Further, while the 43 pumped hydro storage facilities operating in the US today can cumulatively store 553 GWh of energy, a typical salt cavern can store around 200 GWh, meaning a single salt cavern hydrogen storage facility can store significantly more energy.^36^^,^^41^ Although an HDV FC + SCS peaker does not meet the criteria for DAYS storage due to its geographic constraints and cost above $0.05/kWh-cycle, its readiness for near-term commercial deployment, emission-free power generation and potential for future cost reductions and technological enhancements make it an attractive option to consider as an alternative.

### Applying an FC peaker to ERCOT

Here, we demonstrate the value of an HDV FC + SCS peaker within Texas’ EOM region, the Electric Reliability Council of Texas (ERCOT). We choose ERCOT for two reasons. First, as seen in Figure 3, Texas holds a high prevalence of salt caverns suitable for hydrogen storage. These salt caverns are concentrated in the eastern part of the state, in close proximity to major load centers as well as existing power transmission infrastructure which brings wind generation from the western part of the state. Second, ERCOT operates as an EOM, which relies on high forward prices during times of scarcity to incentivize investment in spare generation capacity.^42^ In recent years, both the peaker price threshold as well as the absolute day-ahead peak prices have raised compared to the previous decade (see STAR methods and Figure S1). These factors suggest an FC peaker operating during times of scarcity could provide needed, cost-effective peak power.

We use ERCOT’s historical day-ahead prices from 2011-2022^43^ to assess the potential revenue of an HDV FC + SCS peaker, representing approximately half a system’s economic lifetime of 25 years. We find that a small 1 MW HDV FC + SCS peaker operating at full availability in the top 5% of peak hours over this period would reap 1.8 million US$; if operating in the top 10%, the revenue would increase to 2.1 million US$, quickly recouping the original investment. While this translates to an average of 150 and 175 thousand US$/year, respectively, peak demand events are not evenly distributed over the 12-year period; notably, half of the top 10% of prices from the past 12 years occurred within the last two calendar years, i.e., 2021 and 2022 (see Figure S1). In fact, 2021 accounts for approximately half of the total peaker revenue over this period, driven by markedly high prices on the order of several thousand US$/MWh during Winter Storm Uri in 2021. However, significant revenue would be generated in 2011, 2019, and 2022 as well, due to late afternoon demand spikes and price volatility. This indicates that an FC peaker, depending on the supply/demand conditions and the corresponding market price, could operate both solely for a few hours to serve daily peaks and over multi-day extreme weather events as well. Altogether, however, this irregularity of utilization reflects the unpredictability of peak demand events and supports the deployment of a low-capex peaker capable of flexible operation, like an HDV FC + SCS system.

While Texas leads the US in terms of renewables deployment, ERCOT’s limited interconnections with neighboring grids along with its unique market structure and oversight pose distinct challenges for resource adequacy planners. The grid’s susceptibility to renewable droughts and extreme weather events was notoriously highlighted by Winter Storm Uri in February 2021, which plunged most of the state into blackouts lasting several days, ultimately costing 195 billion US$ in damages.^44^ Leading causes included issues around NG fuel supply^45^ as well as transmission constraints to import power from neighboring states,^46^ which were underscored by Winter Storm Elliott in December 2022.^47^ Although ERCOT has since taken several steps to weatherize facilities^48^ and procure additional reserves outside the market,^49^ these storms have highlighted the value of localized and diversified peak power supply sources that could reliably serve multi-day peak demand events, like fuel cell peakers, further given that high scarcity prices are likely to return with increasingly frequent and severe extreme weather events stemming from climate change. In addition, in the coming years—with the simultaneous growth of new electricity demand as well as clean but intermittent generation sources—the magnitude of the peak power requirement is set to increase, thereby expanding the potential profits of price arbitrage. Further, as Texas develops hydrogen hubs along its Gulf Coast, fuel cell peakers present a complementary opportunity amid plans to construct salt caverns for hydrogen storage and leverage extensive existing energy infrastructure.^50^

## Discussion

Rapid grid decarbonization along with increasing extreme weather threats is playing an increasingly influential role in grid planning. Here, we analyze the techno-economics of a peaker based on HDV FCs that are directly stacked in a stationary application, i.e., without the surrounding vehicle, and paired with salt cavern hydrogen storage; we find that the resulting peaker presents a viable and cost-effective solution for serving peak demand events and supporting power system reliability within the near-term. We find that HDV FC + SCS peakers at present are cost-competitive with NG CTs, and can complement the geographic scope of pumped hydro storage, the only other commercially available emission-free LDES technology.

Provided the availability of geologic hydrogen storage, we foresee a large global potential for these peakers. While domestic IRA incentives contribute to the economic viability of an FC peaker in the US, projected reductions in key cost drivers—including electrolyzers and electricity costs—as well as local market conditions—including higher NG prices or renewable generation policy targets—support the business case for FC peaker adoption on a global scale. For example, another particularly promising location is northwest Europe, where a large salt deposit stretches from the UK across the Netherlands, Denmark, Germany, and into Poland.^51^ While most of the salt caverns here today store NG,^38^ many national governments plan to develop a hydrogen network in part repurposing existing gas infrastructure.^52^^,^^53^ Considering the geographic overlap with the North Sea—where neighboring countries cumulatively target 120 GW of offshore wind capacity by 2030 and 300 GW by 2050^54^—a network of auto FC peakers strategically sited near existing transmission infrastructure could help support wind droughts. Local considerations—such as the European Union’s (EU) plans to roll out subsidies for hydrogen production,^55^ as well as NG prices that have historically typically ranged 2-3x that of the US^56^ and included a carbon price based on the EU Emissions Trading System (ETS)—may help to make auto FC peakers an attractive option. Meanwhile, surveying of salt and rock formations in other major electricity markets around the globe, such as India and China, may further broaden the geographic reach.

Beyond the scope of geologic storage, however, peakers based on HDV FCs and pressurized containers have the potential to provide a location-agnostic peak power solution. While HDV FC + PCS peakers remain cost-prohibitive at present, driven by high levelized storage costs in this low-cycling application, policy support schemes as well as stringent decarbonization targets could act to lower costs and accelerate their deployment. It is forecast that by 2035, the US alone will likely need over 300 GW of peak generation capacity,^6^^,^^7^ while globally, electricity demand is expected to rise by over 250% by 2050.^5^ Given that many municipalities currently plan to retrofit existing fossil fuel CTs or build out new turbines to burn hydrogen as a way of meeting decarbonization goals, hydrogen storage—most likely in the form of pressurized containers—will have to be built out nevertheless. Due to the climate imperative, however, this hydrogen storage can be complemented with auto FCs as not only a zero-carbon, but a zero-emission, solution for peak power generation that is simultaneously cheaper. Together, the dual pursuit of siting automotive FCs with both PCS and geologic storage—which is found in all major US power markets—could see a deployment potential upwards of several hundred GW in the US alone, sufficient to meet the majority of peak power needs in the coming decades.^6^^,^^7^

### Limitations of the study

This paper presents a high-level overview of the feasibility of HDV FC peakers in the US. However, the study is limited in its analysis of an HDV FC peaker’s impact on system operations as well as technical details and schematics of the proposed system. The durability and lifetime of the peaker system is likely to be higher than noted here, given that automotive FCs used in a stationary application would not require the very fast ramp rates necessary for adapting to rapidly changing operational conditions in transportation applications. The study also does not explore location-specific synergies, such as the likely cost savings arising from repurposing salt caverns currently used for NG storage, the co-optimization of the hydrogen production value chain for other end-uses of hydrogen, or the leveraging of ancillary services as an additional revenue source. Further, its reliance on historical data and best-available cost projections does not fully capture the rising uncertainty associated with increasingly volatile energy markets and climate vulnerability. Future work should integrate FC peakers into capacity expansion and system dispatch models to better quantify the value of FC peakers compared to other assets and in a broader amount of locations. The growing deployment of FC peakers could also likely compete with FC demand from the transportation sector, requiring further advancements in manufacturing and the scaling of supply chains associated with FC production.

## STAR★Methods

### Key resources table

**Table:** 

REAGENT or RESOURCE	SOURCE	IDENTIFIER
**Other**		

Costs of wind, solar, battery and fossil technologies	NREL ATB	https://atb.nrel.gov/electricity/2022/index
Costs of fuel cells	US DOE Hydrogen Program	https://www.hydrogen.energy.gov/
Costs of electrolyzers and hydrogen storage	BloombergNEF	https://about.bnef.com/
Duration Addition to electricitY Storage (DAYS) Program Overview	ARPA-E	https://arpa-e.energy.gov/sites/default/files/documents/files/DAYS_ProgramOverview_FINAL.pdf
Historical Day-Ahead Market Prices	ERCOT	https://www.ercot.com/mp/data-products/data-product-details?id=NP4-180-ER
Data generated for this paper	This paper & SI	N/A

### Resource availability

#### Lead contact

Further information and requests should be directed to the lead contact and corresponding author, Amol Phadke (aaphadke@lbl.gov).

#### Materials availability

The study did not generate new materials.

#### Data and code availability

• All data reported in this paper will be shared by the lead contact upon request.
• This paper does not report original code.
• Any additional information required to reanalyze the data reported in this paper is available from the lead contact upon request.

### Method details

#### Fuel cell peaker costs

We estimate the capital costs of a fuel cell peaker by obtaining fuel stack costs from the US Department of Energy (DOE), which vary by fuel cell application as well as annual production capacity.^17^^,^^24^^,^^25^^,^^26^ The remaining integration costs - including the stack Balance of Plant (BOP), electrical Balance of System (BOS), inverter, installation, overhead, and grid interconnection - are approximated based on utility-scale storage system integration costs from the National Renewable Energy Laboratory’s (NREL) Annual Technology Baseline (ATB),^33^ using the 2022 version to obtain 2023 costs (see Table S2). The fuel cell system is assumed to operate at a 10% capacity factor for a lifetime of 30 years; we assume this is sufficient for a Heavy Duty Vehicle (HDV) fuel cell with a durability rating of 25,000 h as well as a stationary fuel cell, yet for Light Duty Vehicle (LDV) fuel cells with a durability rating of 8,000 h, we factor in two stack replacements at present-day costs. Costs are converted to 2023 US$ and the build-up can be seen in Figure 2; to these subtotals of the capital costs, we further apply the 30% Investment Tax Credit from the Inflation Reduction Act to obtain the final fuel cell peaker system capital cost.^22^

The production of green hydrogen - referring to hydrogen that is produced from the electrolysis of water, powered by co-located renewable energy - is then sized assuming the fuel cell system operates at a 10% capacity factor for 30 years, which implies an average of 9 cycles per year with a discharge duration of 100 h. Based on the present-day technical parameters, including a fuel cell efficiency of 54%, storage losses of 0.5%, hydrogen energy content based on the Higher Heating Value (HHV), and an electrolyzer efficiency of 60% (see Table S2),^25^^,^^36^^,^^57^ we find that 1:0.5 ratio is needed between the desired rated power capacity of the fuel cell peaker and the rated power capacity of the electrolyzer, which would only be charged from the co-located renewable generation. From this, we calculate the levelized cost of the electrolyzer based on the annualized capital costs of the electrolyzer, compressor and Balance of Plant (BOP), plus the sum of the Fixed Operating and Maintenance (FOM) expenses as well as Variable Operating and Maintenance (VOM) expenses, the latter of which includes stack replacement costs as well as water (see Table S1).^57^ Electricity costs are derived assuming the electrolyzers are co-located with onshore wind and utility-scale solar PV using US-average capacity factors^58^^,^^59^ and averaging NREL’s levelized costs of electricity for a Class 7 moderate land-based wind and Class 5 moderate utility-scale solar PV,^60^^,^^61^ representing moderate 2023 values. The final electricity price is obtained by further subtracting the renewable energy Production Tax Credit (PTC) of $27.5/MWh for 10 years which is levelized over the system lifetime of 30 years with a 10% discount rate.^22^^,^^62^ Meanwhile, the levelized cost of hydrogen storage (for both salt cavern as well as pressurized container storage) is obtained from Bloomberg New Energy Finance (BNEF), with the cost of compression adjusted with the final electricity price.^36^ The ultimate cost of hydrogen fuel is the sum of the levelized electrolyzer system costs, the final electricity price, and the levelized cost of hydrogen storage, minus the hydrogen PTC of $3/kg for 10 years which is similarly levelized over the system lifetime of 30 years with a 10% discount rate.^22^ All costs are updated to reflect 2023$.

Based on these capital costs and hydrogen fuel costs, the annualized peaker costs are taken to be

$$\text{Annualized}\;\text{peaker}\;\text{cost}\;\left[US\$/\text{kW}-\text{year}\right]=\frac{C\cdot r}{1-{\left(1+r\right)}^{-n}}+\text{FOM}+\text{VOM}+\text{fuel}$$

where C is the capital cost of the fuel cell peaker system, r is the discount rate, n is the system lifetime, FOM are the fixed operating and maintenance costs of the fuel cell system, VOM are the variable operating and maintenance costs of the fuel cell system, and fuel is the annual fuel cost. The annualized peaker cost is a function of the assumed capacity factor, since both the VOM and the fuel costs vary with utilization [Figure 4A].

#### Natural gas peaker costs

The capital costs, fixed operating and maintenance costs, variable operating and maintenance costs, carbon content, and heat rate of the natural gas peaker are based on the 2023 costs of an F-frame CT from NREL’s 2022 ATB.^34^ Meanwhile, the price of natural gas fuel reflects the 10-year Henry Hub spot price average.^63^

Based on these capital costs and natural gas fuel costs, the annualized peaker costs are taken to be

$$\text{Annualized}\;\text{peaker}\;\text{cost}\;\left[US\$/\text{kW}-\text{year}\right]=\frac{C\cdot r}{1-{\left(1+r\right)}^{-n}}+\text{FOM}+\text{VOM}+\text{fuel}$$

where C is the capital cost of the natural gas peaker, r is the discount rate, n is the system lifetime, FOM are the fixed operating and maintenance costs of the natural gas peaker, VOM are the variable operating and maintenance costs of the natural gas peaker, and fuel is the annual fuel cost. The annualized peaker cost is a function of the assumed capacity factor, since both the VOM and the fuel costs vary with utilization [Figure 4A].

#### Sensitivity analysis

We conduct the sensitivity analysis at the point at which the natural gas peaker breaks even with the fuel cell peaker, i.e., a 18% capacity factor in order to assess the impacts of a varying carbon price and natural gas price on the natural gas peaker and the impacts of a varying electrolyzer system capital cost, final electricity price, HDV FC system cost, levelized cost of storage, and discount rate on the fuel cell peaker. The range of values is selected based on either recent historical values or plausible future cost projections out to 2050.

#### Comparison with long-duration energy storage alternatives

We utilize the same assumptions outlined in the Duration Addition to electricitY Storage (DAYS) program, a Department of Energy initiative to develop Long Duration Energy Storage (LDES) technologies which can deliver electricity at a levelized cost of storage (LCOS) of 5 cents/kWh-cycle across the full range of storage durations (from 10 to approximately 100 h at rated power).^12^ This includes a charging electricity price at $25/MWh, 10% discount rate, system lifetime of 20 years as well as the same formula to calculate the levelized cost of storage (LCOS).^12^ We compare the present-day 2023 case with a future best case, which represents an optimistic future scenario without IRA incentives and cost reductions in the electrolyzer capex, salt cavern LCOS, and fuel cell system. Peaker costs utilize the same assumptions as before which are listed in Tables S1 and S2. Results are compared with the DAYS $0.05/kWh-cycle target and pumped hydro storage as presented in the program overview in order to contextualize the costs and benefits of an FC peaker.^12^

#### ERCOT analysis

We obtain publicly available historical day-ahead prices from ERCOT^43^ and consider the range of years with complete data, i.e., 2011–2022, inclusive. We use the hub bus average prices to represent scarcity over the entire ERCOT region. For each calendar year, we sort the data and create a timeseries from maximum to minimum hourly price to create the price duration curves seen in Figure S1. To assess HDV FC + SCS peaker revenue we similarly create a time series, yet over the full 12 years in consideration, and sum the hourly prices in the top 5th and 10th percentiles.
