# Supplementary material for: Leveraging automotive fuel cells can supply zero-emission peak power in the near-term
Source: iScience. 2024 Jun 11;27(7):110246. doi: 10.1016/j.isci.2024.110246 (PMC11246014; doi:10.1016/j.isci.2024.110246)
Supplement: Document S1. Figure S1, Tables S1, and S2 [file mmc1.pdf]

iScience, Volume *27*

Supplemental information

Leveraging automotive fuel cells can supply zero-emission peak power in the near-term

Emilia Chojkiewicz and Amol Phadke

Table S1: List of cost assumptions for hydrogen and natural gas fuel production, related to STAR Methods.

**Variable**	**Unit**	**Value** 2023	**Value** Future best case*	**Note**
System lifetime	years	30	-	
Discount rate	%	10	-	
Target duration per start	hours	100	-	Used to size H2 production
Cycling frequency	cyc/yr	12	-	Used to size H2 production
Wind LCOE [1]	US$/MWh	37.7	-	Class 7 moderate land-based wind, 2023
Solar LCOE [2]	US$/MWh	35.4	-	Class 5 moderate utility-scale solar PV, 2023
Base electricity price	US$/MWh	36.6	-	
IRA RE PTC [3]	US$/MWh	27.5		
Final electricity price	US$/MWh	21.8**	25	
Electricity cost	US$/kg H2	1.21**	1.28	
Capital cost – electrolyzer [4]	US$/kW	1,441	140	Includes stack, BOP, & EPC for PEM
Capital cost – compressor [5]	US$/kW	46	28	
Electrolyzer efficiency [5]	kWh/kg	55	51	Stack & BOP electrical usage
Electrolyzer Fixed O&M [5]	US$/kW/yr	15.6	-	
Electrolyzer Variable O&M [5]	US$/MWh	1.6	-	Includes stack replacement & water costs
Electrolyzer load factor [6,7]	%	55	-	Estimated capacity factor of co-located solar & wind based on US-averages (~40% for wind, ~25% for solar)
Levelized electrolyzer system cost	US$/kg H2	1.70	0.42	
Electrolyzer output pressure [8]	bar	30	-	
Storage pressure [8]	bar	100	-	
Levelized storage cost – salt cavern [8]	US$/kg H2	0.22	0.1	BNEF estimate assuming monthly cycling (20 days fill, 10 days withdrawal) & adjusted with the final electricity price as shown here to reflect the cost of compression to 100 bar for storage
Levelized storage cost – pressurized container [8]	US$/kg H2	6.52	-	
Storage losses [8]	%	0.5	-	
IRA H2 PTC [9]	US$/kg H2	3	-	
Total cost of H2 – salt cavern	US$/kg H2	1.52**	1.81	
	US$/MMBtu	11.31**	13.43	
Natural gas price [10]	US$/MMBtu	3.42	-	Daily Henry Hub spot price average, 2013-2022

* Refers to the most optimistic value that can be reasonably anticipated in the future, either estimated or based on targets provided by the referenced material.
** With IRA PTC levelized over 10 years.
Note: All costs are in 2023 US$.

Table S2: List of cost assumptions for the fuel cell and gas peaker systems, related to STAR Methods.

**Variable**	**Unit**	**Value** 2023	**Value** Future best case*	**Note**
System lifetime	years	30	-	
Discount rate	%	10	-	
Annual capacity factor	%	<10	-	
Target duration per start	hours	<100	-	
H2 energy content [11]	MMBtu/kg	0.1325	-	Higher Heating Value (HHV)
Fuel cell efficiency [12]	%	54	72	
Natural gas heat rate [13]	MMBtu/MWh	9.72	-	F-Frame CT moderate, 2023
Capital cost – LDV FC [14]	US$/kW	97	38	80 kW PEM, stack & BOP, 100,000 units/yr**
Capital cost – HDV FC [15]	US$/kW	235	76	275 kW PEM, stack & BOP, 100,000 units/yr**
Capital cost – stationary FC [5]	US$/kW	1,604	-	1 MW PEM, stack & BOP**
Capital cost – gas turbine [13]	US$/kW	1,006	-	F-Frame CT moderate, 2023
Electrical BOS [16]	US$/kW	176	-	Utility-scale storage in 2021, 4-hour duration
Inverter cost [16]	US$/kW	88	-	Utility-scale storage in 2021, 4-hour duration
Installation cost [16]	US$/kW	79	-	Utility-scale storage in 2021, 4-hour duration
Overhead cost [16]	US$/kW	48	-	Utility-scale storage in 2021, 4-hour duration
Interconnection cost [16]	US$/kW	24	-	Utility-scale storage in 2021, 4-hour duration
System costs subtotal	US$/kW	352	249	
Fixed O&M – FC [5]	US$/kW/yr	15.6	-	Assumed the same for all types of FCs
Variable O&M – FC [5]	US$/MWh	0.6	-	
Fixed O&M – gas turbine [13]	US$/kW/yr	25	-	F-Frame CT moderate, 2023
Variable O&M – gas turbine [13]	US$/MWh	6	-	

* Refers to the most optimistic value that can be reasonably anticipated in the future, either estimated or based on targets provided by the referenced material.
** Excludes stack replacement.
Note: All costs are in 2023 US$.

Figure S1: Price duration curves for ERCOT, related to "Applying an FC peaker to ERCOT" and STAR Methods.

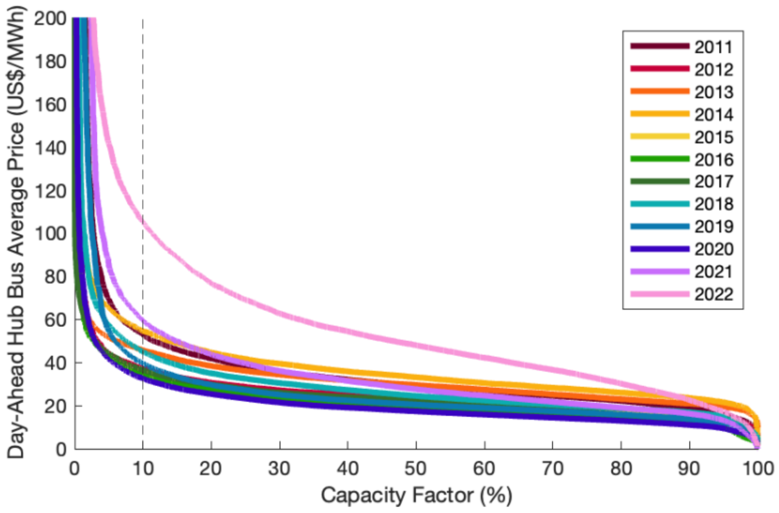

Supplementary References

[1] *Annual Technology Baseline, Land-Based Wind* (NREL, 2022); https://atb.nrel.gov/electricity/2022/land-based_wind
[2] *Annual Technology Baseline, Utility-Scale PV* (NREL, 2022); https://atb.nrel.gov/electricity/2022/utility-scale_pv
[3] *Renewable Electricity Production Credit Amounts for Calendar Year 2022* (Internal Revenue Service, 2022); https://www.irs.gov/pub/irs-drop/a-22-23.pdf
[4] *Electrolysis System Capex by 2050 - Updated Forecast* (BloombergNEF, 2022).
[5] Mongird, K., Viswanathan, V., Alam, J., Vartanian, C., Sprenkle, V., Baxter, R. *2020 Grid Energy Storage Technology Cost and Performance Assessment* (Department of Energy, 2020); https://www.pnnl.gov/sites/default/files/media/file/Final%20-%20ESGC%20Cost%20Performance%20Report%2012-11-2020.pdf
[6] *Land-Based Wind Market Report: 2021 Edition* (Department of Energy, 2021); https://www.energy.gov/sites/default/files/2021-08/Land-Based%20Wind%20Market%20Report%202021%20Edition_Full%20Report_FINAL.pdf
[7] *Southwestern states have better solar resources and higher solar PV capacity factors* (Energy Information Administration, 2019); https://www.eia.gov/todayinenergy/detail.php?id=39832
[8] *Hydrogen: The Economics of Storage* (BloombergNEF, 2019).
[9] *Request for Comments on Credits for Clean Hydrogen and Clean Fuel Production* (Internal Revenue Service, 2022); https://www.irs.gov/pub/irs-drop/n-22-58.pdf
[10] *Annual Henry Hub Natural Gas Spot Price (Dollars per Million Btu)* (Energy Information Administration, 2022); https://www.eia.gov/dnav/ng/hist/rngwhhdA.htm
[11] *Alternative Fuels Data Center Fuel Properties Comparison* (Department of Energy, 2021); https://afdc.energy.gov/files/u/publication/fuel_comparison_chart.pdf
[12] Marcinkoski, J. *DOE Hydrogen and Fuel Cells Program Record #19006: Hydrogen Class 8 Long Haul Truck Targets* (Department of Energy, 2021); https://www.hydrogen.energy.gov/pdfs/19006_hydrogen_class8_long_haul_truck_targets.pdf
[13] *Annual Technology Baseline, Fossil Energy Technologies* (NREL, 2022); https://atb.nrel.gov/electricity/2022/fossil_energy_technologies
[14] Kleen, G., Padgett, E. *DOE Hydrogen Program Record #21001: Durability-Adjusted Fuel Cell System Cost* (Department of Energy, 2021); https://www.hydrogen.energy.gov/pdfs/21001-durability-adjusted-fcs-cost.pdf
[15] Papageorgopoulos, D. *Fuel Cell Technologies Overview* (Department of Energy, 2022); https://www.hydrogen.energy.gov/pdfs/review22/plenary7_papageorgopoulos_2022_o.pdf
[16] *Annual Technology Baseline, Utility-Scale Battery Storage* (NREL, 2022); https://atb.nrel.gov/electricity/2022/utility-scale_battery_storage
